# Flap Complications and Thrombophilia: An Evidence-Based Model and Cost Analysis for Preoperative Screening*

**Published:** 2011-07-08

**Authors:** Kendra G. Bowman, Matthew J. Carty

**Affiliations:** ^a^Department of Surgery, Brigham and Women's Hospital, Boston, MA; ^b^Division of Plastic Surgery, Department of Surgery, Brigham and Women's and Faulkner Hospitals, Boston, MA

## Abstract

**Background:** Preoperative screening for thrombophilias in free flap candidates may be cost-effective. **Methods:** We developed a model for thrombogenic flap complications using reported thrombophilia prevalences and thromboembolic risk ratios, as well as free flap complication rates from our institution. We performed a break-even and incremental cost-effective ratio analysis for several screening and intervention scenarios. **Results:** Our thrombotic free flap complication rate is 4.9%. A full thrombophilia screen breaks even when the cost of complication exceeds $57 000 per patient; a limited screen breaks even at $39 000, and a scenario in which all patients undergo chemoprophylaxis breaks even at $49 000. Incremental cost-effective ratio analyses estimate a cost per avoided flap complication of $33 638 for a full panel scenario, $15 617 for a limited panel scenario and $25 455 for an all therapy scenario. **Conclusions:** Our analyses show that preoperative thrombophilia screening may be a cost-effective measure for the prevention of free flap thrombotic complications.

Free tissue transfer is a commonly performed reconstructive surgical procedure. An estimated 19 000 microsurgical free tissue transfers were performed during 2008 in the United States for a broad range of reconstructive indications.^1^ Free flap complications are expensive, morbid, and psychologically stressful for the patient and equally disconcerting for the surgeon. Identification of preoperative patient risk factors may facilitate prevention of intraoperative and postoperative adverse events.

Thrombosis represents a significant source of flap complications and may lead to reoperation, extended hospital stays, flap necrosis, and loss. For all types of free flaps, reported thrombotic flap complication rates requiring reoperation range between 3% and 12%.^2^^-^9 Thrombotic events are typically attributed to mechanical factors; however, several case reports of thrombophilia associated with flap failure have been reported,^10^^-^14 and thrombophilias in free flap patients have received recent attention in the literature.^12^,^15^ The association between thrombotic flap complications and thrombophilias has not been rigorously studied. A few small investigations reported conflicting findings, but none have been adequately powered to establish causal effect.^16^,^17^ However, acquired and genetic thrombophilias affect approximately 25% of the population^18^ and are known to contribute to thrombosis in a variety of settings. Taken together, these observations point to a potentially significant relationship between thrombophilias and thrombotic flap complications.

Current knowledge of the relative risks for thrombosis imposed by thrombogenic conditions comes from studies on venous thromboembolism (VTE) and arterial thrombosis in normal and thrombophiliac cohorts. In patients with known thrombophilia, a 1.7- to 11-fold annual relative risk of initial VTE is observed compared to normal counterparts.^18^^-^21 This corresponds to an annual incidence of initial VTE of 0.25% to 1.9% for patients with thrombophilia compared with 0.05% annual incidence for normal populations. The role of thrombophilias in arterial thromboembolic events is less well established but is associated with antiphospholipid syndrome^14^,^22^ and hyperhomocysteinemia,^23^ but not consistently with other thrombophilias.

Venous thromboembolism incidence is increased in patients with malignancy,^24^ and in trauma patients,^25^ conditions often found in free tissue transfer candidates. In addition, prospective studies of VTE in asymptomatic thrombophiliacs evidence that more than half of first VTE events occur in association with trauma, surgery, or puerperium.^20^,^26^ Taken together, it is plausible that thrombophilias contribute to thrombotic complications in microvascular surgery, and that undiagnosed thrombophilias underlie a greater proportion of flap complications than is currently recognized.

We sought to develop a model to estimate the cost-effectiveness of preoperative thrombophilia screening for patients undergoing free tissue transfer. The risk of thrombosis attributable to a thrombophilia during free flap surgery is not known, and the relative risk ratios for thrombophiliacs versus normal cohorts have not been reported. We posit that rational proxies for the relative risks posed by thrombophilias in the setting of microvascular surgery are those known for initial VTE events. This model assumes that the risk of thrombosis posed by microsurgery for both normal and thrombophiliac cohorts is comparable to the annual risk of a first VTE. Accordingly, for each of the inherited thrombophilias, we identified the reported risk ratio for an initial VTE for previously undiagnosed subjects.^18^,^21^ In conjunction with the known prevalences of each thrombophilia, the proxy relative risks allow estimation of the number of thrombotic flap complications that are attributable to thrombophilia that would, in turn, be potentially amenable to prophylactic interventions.

This rational and evidence-based approach allows exploration of the conditions under which preoperative thrombophilia screening may be cost-effective. With the incorporation of financial data, this model enables assessment of the costs of screening and prophylactically treating free tissue transfer patients relative to the costs of current baseline practice. For cost-effectiveness analyses, we used a break-even analysis and incremental cost-effectiveness ratio (ICER). The *break-even point* is defined as the point at which a variable, such as complication cost, renders the cost of an intervention scenario equal to the cost of the baseline scenario. We applied the break-even analysis to determine the flap complication cost at which an intervention scenario and the baseline scenario break even in cost. The ICER is the ratio of the difference in costs between therapeutic interventions to the difference in effects between interventions. An ICER is expressed in dollars per unit of health gained, and ICER analysis is used in health care economics to determine the additional cost per unit of health benefit gained when comparing one medical intervention with another. Each metric offers distinctive and informative insights on cost-effectiveness, thus we incorporated both in the evaluation of various scenarios in our analysis.

## PATIENTS AND METHODS

We retrospectively reviewed all free tissue transfers performed by faculty within the Division of Plastic Surgery at Brigham and Women's Hospital over the period October 2004 to October 2009. We identified cases in which reoperation was performed for vascular compromise of the flap. A *thrombotic complication* was defined as intraoperative documentation of idiopathic thrombosis; specifically, we excluded cases in which a mechanical cause of thrombosis was evident. From these data, we calculated a thrombotic complication rate for our free flap series (Table 1). We reviewed patient billing records for the cases of thrombotic complications and matched a control group of uncomplicated free flaps by indication, year, and flap type. To establish the average cost of thrombotic flap complications, the direct costs for each operative admission period were compared between the 2 groups (Table 2).

We reviewed the literature on thrombophilia and identified those conditions that are most commonly implicated in clinical disease. As a proxy for the relative risk of thrombosis imposed by a thrombophilia in the perioperative period, we turned to the known risk ratios for initial venous thromboembolism for each of the thrombophilias. In each case, we chose the most conservative established risk ratio. Using values from our institution's laboratories, we then calculated the cost of screening for these conditions and ascertained the sensitivities and specificities of each test (Table 3). We determined the cost of in- and outpatient enoxaparin therapy for 1 month at our institution.

Using these data, we developed a mathematic model to estimate the number and cost of anticipated thrombotic complications for 4 scenarios, each with a cohort of 10 000 patients. The *Baseline Scenario* included the total number and cost of flap complications with no preoperative screening. The *Full Panel Scenario* examined the total number and cost of flap complications, in addition to the cost of preoperative screening for every patient for all thrombophilias and intervention for test-positive patients with 1 month of prophylactic enoxaparin. We assumed 88% effectiveness of thrombosis prophylaxis based upon reported figures from a meta-analysis.^28^ As a conservative measure, we limited the model's calculation of risk of thrombosis to 50% for each thrombophilia. The *Limited Panel Scenario* described a lower-cost screen for a subset of thrombophilias that the model predicted would capture 87% of the disease-positive patients. Assays were included on the basis of the number of test-positive patients predicted by the model, which was a function of each condition's prevalence, relative risk, and test sensitivity. Our analysis was structured to account for thrombotic events expected in patients suffering from thrombophilias excluded from the limited panel (Table 3). Finally, the *All Therapy Scenario* assessed a circumstance in which no preoperative screen was performed but all patients underwent prophylactic enoxaparin therapy. We then compared the total cost and the absolute difference in flap failures of each intervention scenarios relative to the baseline and calculated a break-even point and ICER for each comparison. The parameters used in the predictive model, break-even, and ICER analyses are shown in Table 4.

## RESULTS

We reviewed 264 free flaps at our institution performed over a 5-year period. Thrombosis-related complications occurred in 13 (4.9%) of flaps (Table 1), leading to 5 flap failures (1.9%). Billing records showed an average cost of flap complication over a case-controlled uncomplicated flap of $23 246 (Table 2).

Through review of the literature, we identified the 9 most common thrombophilias as factor VIII excess, activated protein C resistance, hyperhomocysteinemia, anticardiolipin antibody, lupus anticoagulant, prothrombin G20210A mutation, protein S deficiency, antithrombin deficiency, and protein C deficiency. We applied the known relative risks for initial VTE (range, 2.5–11) for these conditions as proxy relative risks for thrombotic microsurgical complications (Table 3). Our institutional laboratory cost for a complete thrombophilia screening panel was $1206 per patient, while a limited panel of the 5 thrombophilias predicted to cause 87% of complications was $572 per patient. The limited panel includes activated protein C resistance, lupus anticoagulant, hyperhomocysteinemia, protein S deficiency, and factor VIII excess. Using reported thrombophilia prevalences and risk ratios and our complication rate of 4.9%, our model estimated that 73% of flap thromboses are attributable to thrombophilia, or an absolute rate of 3.6% of cases.

Assuming a theoretical cohort of 10 000 free flap candidates and applying known prevalence and screening test sensitivity statistics for each of the included thrombophilias, our model predicted a total of 2889 patients who would test positive for hypercoagulable conditions in a preoperative screening panel. We assumed that no operative intervention would be cancelled because of a positive screening test and that thrombosis prophylaxis would consist of a thirty-day course of enoxaparin costing $2100 per patient. The efficacy of prophylactic therapy in such cases was assumed to be 88%, pursuant to prior validated studies.

**Figure 1:**
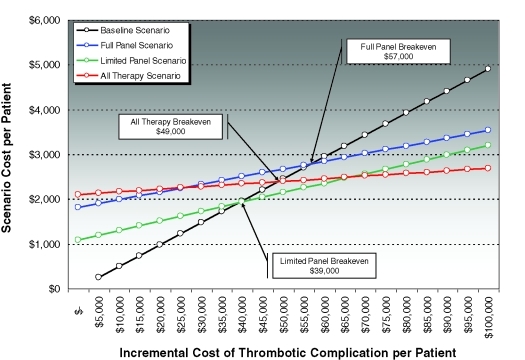
Break-even analysis demonstrates the break-even points for the 3 intervention scenarios compared to baseline.

Break-even analyses demonstrated that a complete preoperative thrombophilia screen (*Full Panel Scenario*) breaks even when the cost of thrombotic flap complication exceeds $57 000 per patient. A limited thrombophilia screen that captures 87% of anticipated thrombotic complications at a substantially reduced panel cost (*Limited Panel Scenario*) breaks even when the cost of thrombotic flap complication exceeds $39 000 per patient. Finally, prophylactic treatment of all patients with 1 month of enoxaparin therapy in the absence of any preoperative screening (*All Therapy Scenario*) breaks even when the cost of thrombotic flap complication exceeds $49 000 per patient (Table 5, Fig 1).

ICER analyses performed for each of the intervention scenarios compared to the baseline scenario. For the *Full Panel Scenario*, the cost per avoided flap complication was $33 638. For the *Limited Panel Scenario*, the cost was $15 617 per avoided thrombotic flap complication, and in the *All Therapy Scenario*, the cost was $25 455 per avoided thrombotic flap complication (Table 5).

## DISCUSSION

Anastomotic thrombosis is a principal concern in free flap reconstructive surgery, and efforts to limit this complication justify many of the care measures routinely employed in the care of patients undergoing these procedures. Although thrombosis is sometimes secondary to mechanical factors including excessive vessel stretch or kinking, arterial and/or venous thrombotic occlusion often occurs in the absence of any apparent technical concerns. Increasing recognition of this point has recently led to heightened investigation of the potential role of thrombophilias in free flap surgery. The prevalence of thrombophilias in the general population is approximately 15%, and thrombophilias have a well-established causal relationship to venous thromboembolic events. While the contribution of thrombophilia conditions to thrombosis is unknown, it is likely that thrombophilias play a significant role in free flap thrombotic complications that is under recognized.

Our study represents the first attempt to ascertain whether preoperative screening and perioperative treatment for previously undiagnosed thrombophiliacs undergoing free flap procedures may be a cost-effective measure. Our theoretical model estimates that thrombophilias may account for up to 73% of nonmechanical thrombotic free flap complications, based on an overall institutional nonmechanical thrombotic free flap complication rate of 4.9%. It furthermore suggests that the cost of preoperatively screening all free flap candidates and prophylactically anticoagulating those who demonstrate tests positive for thrombophilia conditions breaks even with status quo practice when the incremental cost of thrombotic flap complication per patient exceeds $39 000 (for a limited screening panel) and $57 000 (for a full screening panel). As an alternative to both preoperative screening and the status quo, we also estimated the break-even point for presumptively anticoagulating all free flap candidates, which, at $49 000, fell roughly midway between the limited and full-screen watershed marks.

These findings are notable, given their implications for both the scope and impact of thrombophilia conditions relative to free flap surgery. If thrombophilias truly account for nearly three quarters of thrombotic flap complications, efforts to identify and treat free flap candidates with these conditions have the potential to significantly reduce perioperative morbidity in this patient cohort. What's more, doing so may come at an overall cost that is roughly equivalent to status quo practice—an observation that is particularly salient, given increasing national attention on identifying and enacting medical practices deemed to be cost-effective.

The utility in performing theoretical cost analyses requires comparing their results to real cost data. While our break-even assessments provide an indication as to when our posited intervention scenarios achieve efficiency equivalent to the status quo, the question remains: what is the actual incremental cost of a thrombotic free flap complication? Toward this end, we have offered the results of a preliminary cost comparison analysis that provides an initial approximation of $23 000 per patient. This assessment is admittedly rudimentary and does not likely capture the full scope of incremental costs associated with thrombotic flap complications; we expect more robust analyses performed in the future to demonstrate a substantially higher value.

Providing this preliminary cost estimation does, however, allow us to begin to frame discussions regarding preoperative screening and therapy relative to current practice in a rational and meaningful manner. First, it permits the establishment of a benchmark against which to judge the break-even analyses of our intervention scenarios. This, in turn, allows us to tackle the question: how much will screening and treating free flap candidates cost relative to baseline? Our present analyses suggest a $14 000 to $34 000 per patient differential between break even and baseline; with further investigation of direct costs, however, we expect this differential to markedly reduce and, perhaps, cross the break-even threshold. Second, it affords the capacity to assess the marginal utility of increased cost relative to expected benefit in the form of ICER analyses. This, in contrast, allows us to tackle the question: how much expense is required to avert a single thrombotic flap complication? Our ICER estimations demonstrate incremental cost per avoided thrombotic flap complication results ranging from $15 617 for the *Limited Panel Scenario* to $33 638 for the *Full Panel Scenario*. These results illustrate the augmented cost-effectiveness of targeted screening and treatment efforts and, as above, will likely decrease as more reliable cost data is derived.

For the purposes of our study, it is assumed that a positive thrombophilia screening test result for a given free flap candidate would result in a change in baseline practice. Within our institution, enoxaparin therapy is endorsed by hematologists as the optimal prophylactic treatment regimen in free flap patients who are known thrombophiliacs and was therefore assumed by us to be the intervention of choice for modeling purposes. While general agreement regarding the choice of enoxaparin exists, the duration of therapy remains debatable. Conservative hematologists within our institution favor a 30-day course of prophylactic therapy, yet a growing number of specialists have begun to argue for more limited (2 week) regimens. Although our model assumes the more conservative 30-day regimen, substitution with a 2-week therapy course would lead to a substantial reduction in our cost analyses that would further favor the adoption of preoperative screening and therapy practices. In addition, it is possible that the employment of alternative therapeutic interventions for local anticoagulation (eg, flap-directed heparinization or tissue plasminogen activator administration) would offer lower-cost options of equivalent efficacy that would further influence our analyses.

There are several limitations of this study that must be acknowledged:

1. The determination of our institutional thrombotic flap complication rate was predicated upon the identification of problematic cases based on the subjective assessment of the operative surgeon as reflected in operative note. No objective means of confirming the assignation of a case as being of mechanical or nonmechanical thrombotic etiology was possible.
2. Our analytic model is driven off of multiple assumptions regarding thrombophilia conditions and therapeutic efficacy that, while data-supported, remain open to debate. In particular, the use of the relative risk of initial VTE as a proxy for the relative risk of thrombotic complication for free flap surgery may be an inaccurate assumption, and further studies are warranted to determine the precise role of thrombophilias in the setting of microsurgery. In addition, the potential for simultaneous coexistence of multiple thrombophilias in the same theoretical patient is not captured, as little data regarding coincidence rates of thrombophilias exists.
3. As described earlier, our institution-specific incremental cost of thrombotic flap complication calculation is admittedly rudimentary and deserves more detailed evaluation. However, the current analysis likely underestimates the full incremental cost and, therefore, likely understates the efficiency and cost-effectiveness of intervention.
4. Our model excludes the cost of potential bleeding complications from administration of therapeutic enoxaparin. However, a meta-analysis for extended enoxaparin prophylaxis found no increase for pelvic and abdominal surgeries,^28^ and other studies have found the rate of hematomas and postoperative bleeding to be small or not significant relative to controls. Thus, the best data for added complications from prophylaxis would have a small to negligible influence on this model would and would not statistically influence the estimates.
5. In accord with studies on VTE prophylaxis in other cohorts, in our model, we assume 88% efficacy of therapeutic enoxaparin for those who test positive for thrombophilias. The effectiveness of therapeutic anticoagulation may not be borne out in the setting of microsurgery, and clinical trials are needed.

In light of these limitations, we submit this study as a means to begin, rather than conclude, discussions regarding the potential cost-effectiveness of preoperative screening and perioperative treatment for previously undiagnosed thrombophiliacs undergoing free flap procedures. Future studies directed at further elucidating the true thrombotic flap complication rate and marginal cost of thrombotic flap complication are clearly warranted, as are investigations to validate the use of VTE relative risks as proxy measures for specific thrombophilia conditions. We look forward to collaborating on such efforts in the future.

## Figures and Tables

**Table 1:** Institutional Free Flap Analysis*

Flap Type	Total Flaps (A)	%	Thrombotic Complications (B)	%	% Thrombotic Complications (A/B)
ALT	28	10.6	1	7.7	3.6
DIEP	121	45.8	1	7.7	0.8
Face	1	0.4		0.0	0.0
Fibula	7	2.7	1	7.7	14.3
Gracilis	10	3.8		0.0	0.0
Latissimus dorsi	4	1.5	1	7.7	25.0
VRAM	18	6.8	1	7.7	25.0
RFF	52	19.7	2	15.4	3.8
Scapula	4	1.5	2	15.4	50.0
SGAP	5	1.9	2	15.4	40.0
SIEA	7	2.7	2	15.4	28.6
TRAM	6	2.3		0.0	0.0
Vastus lateralis	1	0.4		0.0	0.0
Total	264	100.0	13	100.0	4.9

* ALT indicates anterolateral thigh flap; DIEP, deep inferior epigastric perforator flap; RFF, radial forearm flap; SGAP, superior gluteal artery perforator flap; SIEA, superficial inferior epigastric artery perforator flap; TRAM, transverse rectus abdominis myocutaneous flap; VRAM, vertical rectus abdominis flap.

**Table 2:** Summary of Observed Thrombotic Complications and Direct Cost Comparison

	Thrombotic Complication Index Cases				Uncomplicated Comparator Cases			
Flap Type	Year	Thrombotic Complication	Indication	Direct Cost	Year	Indication	Direct Cost	Differential
ALT	2005	Flap loss	Chronic lower extremity wound	$70 045	2005	Chronic lower extremity wound	$68 667	$1 378
DIEP	2009	Venous thrombosis	Breast cancer	$44 880	2009	Breast cancer	$18 748	$26 132
Fibula	2006	Venous thrombosis	Traumatic facial wound	$47 205	2007	Traumatic facial wound	$16 500	$30 705
Latissimus dorsi	2008	Flap loss	Head and neck cancer	$103 015	2006	Head and neck cancer	$17 578	$85 437
Latissimus dorsi	2005	Flap loss	Traumatic lower extremity wound	$14 718	2005	Head and neck cancer	$16 299	($1581)
VRAM	2008	Flap loss	Traumatic lower extremity wound	$55 591	2008	Chronic lower extremity wound	$24 198	$31 393
RFF	2004	Venous thrombosis	Intraoral cancer	$50 426	2004	Intraoral cancer	$30 643	$19 783
RFF	2009	Venous thrombosis	Intraoral cancer	$75 751	2009	Intraoral cancer	$28 891	$46 860
Scapula	2005	Arterial thrombosis	Romberg's disease	$44 474	2008	Thermal injury	$29 335	$15 139
SGAP	2008	Flap loss	Breast cancer	$21 418	2008	Breast cancer	$23 492	($2 074)
SGAP	2008	Venous thrombosis	Breast cancer	$38 004	2008	Breast cancer	$18 751	$19 253
SIEA	2008	Arterial thrombosis	Breast cancer	$43 390	2009	Breast cancer	$45 304	($1 914)
SIEA	2008	Venous thrombosis	Breast cancer	$51 963	2009	Breast cancer	$20 277	$31 686
Average				$50 837			$27 591	$23 246

* ALT indicates anterolateral thigh flap; DIEP, deep inferior epigastric perforator flap; RFF, radial forearm flap; SGAP, superior gluteal artery perforator flap; SIEA, superficial inferior epigastric artery perforator flap; VRAM, vertical rectus abdominis flap.

**Table 3:** Thrombophilia Panel Detail*

Condition	Prevalence,^27^ %	Prevalence in, Patients With 1st VTE, %	Relative Risk for 1st VTE^18^,^21^	Test Sensitivity,^18^,^21^ %	Predicted Test Positive Patients	Full Panel Cost†	Limited Panel Cost†
Activated protein C resistance	5.00	20-50	7.5	100	68	$116	$116
Anticardiolipin antibody	2.50	12	3.2	95	13	$222	
Antithrombin deficiency	0.17	1-5	28.2	100	9	$96	
Factor VIII excess	11.00	25	7.1	100	141	$154	$154
Hyperhomocysteinemia	5.00	10-25	2.5	100	23	$137	$137
Lupus anticoagulant	2.50	12	11.0	100	45	$49	$49
Protein C deficiency	0.14	3-9	24.1	100	6	$114	
Protein S deficiency	0.70	2-8	30.6	100	35	$116	$116
Prothrombin G20210A mutation	2.00	6	5.2	100	19	$202	
Total					358	$1206	$572

*VTE indicates venous thromboembolism.

†Partners Healthcare Clinical Laboratory.

**Table 4:** Model Assumptions

Model Variable		Assumed Value
Patient population		10 000
Flap cancellation rate		0%
Cost of screening panel*	$	1206
Cost of anticoagulation therapy per patient	$	2100
Efficacy of anticoagulation therapy		88%
Cost of thrombotic complication per patient*	$	23 246
Thrombotic complication rate		4.9%

*Variable adjusted in cost-efficiency break-even analysis

**Table 5:** Summary Results of Scenario Analyses*

Scenario	Cost per Patient		Thrombotic Complications	Break-Even Point		ICER	
Baseline	$	1067	490	N/A		N/A	
Full panel	$	2186	171	$	57 000	$	33 638
Limited panel	$	1542	212	$	39 000	$	15 617
All therapy	$	2228	59	$	49 000	$	25 455

*ICER indicates incremental cost-effectiveness ratio. Intervention scenario results are relative to baseline scenario.
